# A comparison of information sharing behaviours across 379 health conditions on Twitter

**DOI:** 10.1007/s00038-018-1192-5

**Published:** 2018-12-26

**Authors:** Ziqi Zhang, Wasim Ahmed

**Affiliations:** 1 University of Sheffield, Sheffield, UK; 2 Northumbria University, Northumbria, UK

**Keywords:** Public health, Health conditions, Social media, Twitter, Network analysis, Data science

## Abstract

**Objectives:**

To compare information sharing of over 379 health conditions on Twitter to uncover trends and patterns of online user activities.

**Methods:**

We collected 1.5 million tweets generated by over 450,000 Twitter users for 379 health conditions, each of which was quantified using a multivariate model describing engagement, user and content aspects of the data and compared using correlation and network analysis to discover patterns of user activities in these online communities.

**Results:**

We found a significant imbalance in terms of the size of communities interested in different health conditions, regardless of the seriousness of these conditions. Improving the informativeness of tweets by using, for example, URLs, multimedia and mentions can be important factors in promoting health conditions on Twitter. Using hashtags on the contrary is less effective. Social network analysis revealed similar structures of the discussion found across different health conditions.

**Conclusions:**

Our study found variance in activity between different health communities on Twitter, and our results are likely to be of interest to public health authorities and officials interested in the potential of Twitter to raise awareness of public health.

**Electronic supplementary material:**

The online version of this article (10.1007/s00038-018-1192-5) contains supplementary material, which is available to authorized users.

## Introduction

With the exponential growth of Web 2.0 and the increasing uptake of social media platforms, Twitter has become the most popular channel for online communication and engagement of public health matters (Thackeray et al. 2012). Twitter is a social networking and microblogging platform where users post and interact with messages, or ‘tweets’. Twitter enables its users to engage in effective and real-time information sharing and dialogic relationship building with each other (Park et al. 2016), through its various interactive features.

Due to the potential of Twitter to provide insight into public views and opinions related to health and the ability to retrieve data at little cost, it has become a valuable resource for research (Moorhead et al. 2013). Current research typically examines communities interested in specific health conditions, often identified by particular users, or the use of certain keywords (e.g. dementia) or hashtags (e.g. #autism) in the tweets. Using Twitter’s search function, researchers collect tweets by matching certain keywords or hashtags with tweets restricted to certain time frames, to create topic-specific Twitter database for analysis. Based on the types of research questions, current research can be roughly split into *three groups*, while the majority of these utilise quantitative data mining techniques with fewer using qualitative approaches (Ahmed 2018).

The first group applies *data mining to Twitter streams* to discover novel patterns that can predict future events or enhance our existing knowledge. These include studies that used Twitter for public health surveillance (Szomszor et al. 2010; Zhang et al. 2017), research that focused on topic and theme mining (Paul and Dredze 2011) and information extraction for pharmacovigilance (Curtis et al. 2017; Ginn et al. 2014). The second group concerns *analysing the nature* (e.g. *content, quantity*) *of information sharing* concerning particular health conditions on Twitter, often by studying variables such as the number and demographics of users, network structure, tweeting frequency, topics indicated by keywords and hashtags, and the geographic and temporal dynamics of the tweeting behaviours. Existing work has mostly focused on different types of cancer. Some studied tweeting behaviours of health organisations (Thackeray et al. 2012), or individual users (Borgmann et al. 2016; Salem et al. 2016; Sinnenberg et al. 2016; Pemmaraju et al. 2017); some analysed the shared content (Tsuya et al. 2014; Rosenkrantz et al. 2016; Xu et al. 2016; Loeb et al. 2017). The third type of research examines the *impact of content sharing* in terms of engaging audience and growing communities (Ferguson et al. 2014; Singh and John 2015; Brady et al. 2017; Rabarison et al. 2017; Ahmed et al. 2018) or providing emotional support to patients (Pagoto et al. 2014; Reavley and Pilkington 2014). For example, Reavley and Pilkington (2014) showed Twitter to be an effective channel to obtain emotional support on certain mental health-related issues.

However, previous work has been limited in two ways. *First*, they have typically focused on a single, or a very limited set of related health conditions, usually different kinds of cancers. Very little work has compared Twitter communities of different health conditions which could uncover potential trends and highlight popular health topics. Furthermore, certain scholars have noted an imbalance in regards to the amount of attention certain health conditions may receive on Twitter (Sajadi and Goldman 2011). For example, Loeb et al. (2017) highlighted that the breast cancer community is more than three times larger than prostate cancer (by users) and has ‘a seven-fold higher activity’ (by tweets), although prostate cancer has a higher mortality rate in, for example, the UK (The Guardian 2018).

*Second*, although many studies quantified the presence of particular communities on Twitter using a range of variables, to the best of our knowledge no work has attempted to understand the interdependence among these variables and in particular, whether it is possible to identify the relationship between the level of engagement in a community and other observable variables (e.g. how users tweet). This can be very useful because it can be argued that the engagement in a community is crucial for its growth (Park et al. 2016), and a highly engaging community may not depend on its size or the popularity of the health condition it represents.

This research conducted a comparative analysis of 379 communities totalling over 1.5 million tweets and 450,000 users related to different health conditions using a multifacet and multivariate model. Community was evaluated from three facets including the level of engagement, user and content characteristics. Each facet is described by multiple quantifiable variables. Our hypothesis was that a highly engaging community potentially attracts more audience, and therefore, the engagement in the community can be essential for growing and raising awareness of a community. More importantly, it is possible to ‘influence’ engagement by proactively exercising control over other factors from user and content aspects, such as encouraging content creation or creating content in certain ways. We conducted correlation analysis to discover interesting dependences between engagement and other facets and utilised social network analysis to identify the typical structure and interaction within some typical communities.

Our findings contribute to the research and practice of public health in two ways. First, we quantified and discovered different levels of engagement in various health conditions. This will enable an evidence-based approach for public health authorities, researchers and practitioners to effectively identify and target conditions that can benefit from intervention, e.g. via awareness campaigns. Second, we discovered controllable factors that can potentially affect levels of engagement in these communities. This will enable more effective communication during the intervention.

## Method

Our method first collects tweets potentially discussing different health conditions and then applies the multifacet and multivariate modelling to each health condition based on the tweets and users found for that condition. This creates a dataset of health conditions each represented by a list of numeric variables. We then conduct correlation analysis to the data. From the findings of this analysis, we select a sample of conditions for network analysis.

### Data collection

Following standard practice from previous work, we first defined the presence of a community on Twitter based on the usage of hashtags in tweets. We used a collection of disease hashtags compiled by the Symplur Healthcare Hashtag Project. These hashtags are filtered by Symplur and are believed to represent different diseases and conditions (e.g. #Colitis). However, not every hashtag represents a unique disease (e.g. #Allergy), and some diseases can have multiple hashtags (e.g. #Diabetes, #Diabetic). Thus we manually cleaned these hashtags by removing those that were too generic (e.g. #NotJustOneDisease) or indicative of symptoms rather than diseases (e.g. #Overweight) and reorganised the remaining hashtags into 379 different diseases they represent (to be referred to as ‘disease communities’ on Twitter). This process was carried out by two researchers in a double-confirmation process. Although both were non-clinicians, each hashtag was already supplied with a textual description by Symplur to identify the associated disease. In rare cases where they cannot decide, they searched the hashtags on Twitter and used the tweets to support their interpretation.

Next, the Twitter Application Programming Interface (API) was utilised to collect real-time tweets that contained at least one of these hashtags for a period of 1 month between April and May 2018. In addition to a tweet’s text, metadata such as the hashtags, URLs, likes and retweet counts were also collected. This created a dataset of around 1.5 million tweets, from which we derived over 450,000 users.

### The multifacet and multivariate modelling of a community

In this step, we used a list of variables to describe each disease community identified before. We calculated the values of these variables for each community, based on its tweets and users. We believe that some of these variables could be reliable measures while others as predictors of engagement in a community. To discover such relationships, we proposed to measure a community’s Twitter presence by *three facets*, each defined with a number of variables summarised in Table 1.

**Table 1:** The proposed facets and variables by this study to model a Twitter online community

Facet	Variable	Explanation
Level of engagement	%Retweet	Percentage of tweets that have been retweeted
	Mean retweet freq	Average frequency of retweets for all retweeted tweets
	%Like	Percentage of tweets that have been liked
	Mean like freq	Average number of likes for all tweets
	%Reply	Percentage of tweets that reply to other tweets
	%Quote	Percentage of tweets that quote other tweets
User characteristics	Total users	Number of unique users that have tweeted during data collection
	%New content creator (NCC)	Percentage of users that have created new tweets (i.e. excl. retweet)
	%Content propagator (CP)	Percentage of users that have retweeted existing tweets
	Mean NCC new tweets (NT)	Average number of new tweets per NCC
	Mean CP retweets (RT)	Average number of retweets per CP
	%NCC-outlier by NT	Percentage of NCCs that are outliers (detected using the IQR method) who created too many new tweets
	%CP-outlier by RT	Percentage of CPs that are outliers who retweeted too often
	Mean NCC followers	Average number of followers per NCC
	Mean CP followers	Average number of followers per CP
	%NCC-outlier by followers	Percentage of NCCs that are outliers who have too many followers
	%CP-outlier by followers	Percentage of CPs that are outliers who have too many followers
Content characteristics	Total tweets (new and RT)	Number of total tweets collected, including new tweet and retweet (same for the following)
	Mean hashtags	Mean number of hashtags per tweet
	Mean mentions	Mean number of user mentions (e.g. ‘@BBC’) per tweet
	Mean URLs	Mean number of URLs per tweet
	Mean media	Mean number of media data (e.g. image, video) per tweet
	Mean unique words	Mean number of unique words per tweet
	Mean length	Mean number of words per tweet

Specifically, *level of engagement* describes the extent to which users in a community interact with the content. In addition to percentages of retweets and likes that are used in previous work, we also calculate the frequency of retweets and likes, as well as percentage of replies and quotes. Intuitively, the higher these numbers, the more interaction is observable in a community.

*User characteristics* describe user properties or behaviours, such as users that created new tweets (NCC, see Table 1) as opposed to those only retweeted (CP), frequency of their content creation or propagation (i.e. retweet), their followers and the percentage of users whose behaviours deviate significantly from the population in that community. For this, we use the well-known interquartile range (IQR) method for detecting outliers in a distribution. Given a variable and the set of its values, we identify the percentage of values that are more than 1.5 times of the upper quartile value. IQR is known to be less sensitive than the standard deviation-based method, which often overreacts to outliers in a distribution. We applied this method to detect the users who created too many new tweets, retweeted too often, and who have significantly more followers compared to other community users. The idea is to investigate whether it is possible to alter user behaviours or identify those with certain behaviours to influence engagement.

*Content characteristics* describe observable metadata associated with tweets, such as the use of hashtags and media. The idea is to study whether certain ways of content creation can attract more interaction.

### Correlation analysis

In this step, we took the calculated variables for all disease communities above and conducted two kinds of correlation analysis to discover dependencies between individual variables and between facets. We first applied a ‘pre-processing’ to the data by excluding ‘total users’ and ‘total tweets’ because we want to study these communities regardless of their sizes. Also we excluded communities that are extremely small—those that have less than 100 tweets or 50 users—because in these cases, the statistics calculated would be unreliable. This left a total of 291 communities for this part of analysis. Further, we apply ‘standardisation’ to each variable, by subtracting the mean from each value, and divide the difference by the standard deviation. This is the typical procedure in statistical analysis to ensure variables of different scales (e.g. percentage vs. integers) are normalised such that they are comparable.

Our first analysis computes pairwise Pearson correlation between any one of the six engagement variable with any single variable from the other two facets. The goal is to discover potential dependence between any single aspect of the user or content with any single aspect of the community engagement. However, in some cases, the dependency relationship we aim to discover can be very complex and only exists between certain combinations of variables. For example, our lifestyle variables such as alcohol consumption, exercise and reading habits will relate to our risks of developing diseases such as diabetes, dementia, cancer and heart disease. But we may find the strongest correlation exists between our alcohol consumption and exercise, and our chance of developing diabetes and heart disease.

Our second analysis uses *Canonical Correlation Analysis (CCA)* to discover such ‘complex’ correlations between two sets (i.e. facets) of variables, i.e. user or content characteristics and engagement. Given two sets of variables $X = \left\{ {x_{1} ,x_{2} , \ldots ,x_{i} } \right\}$ and $Y = \left\{ {y_{1} ,y_{2} , \ldots , y_{j} } \right\}$, CCA hypothesises each set to be described by a ‘latent variable’ (called *canonical variate*) *CX* and *CY* which is a linear combination of all variables in the set with different weights (called ‘canonical weights’), e.g. $CX = a_{1} x_{1} + a_{2} x_{2} + \cdots + a_{i} x_{i}$. In an analogy, *CX* and *CY* are ‘composite index’ that integrate all variables from each set. Then depending on the setting of these canonical weights (i.e. $a_{i}$), we can measure different correlations between *CX* and *CY*. The problem therefore resolves to optimise the canonical weights for the two sets of variables in order to maximise the correlation between *CX* and *CY*, which is the ‘canonical correlation’. A high absolute value of canonical correlation indicates strong dependence between the two sets of variables. Then the significance of each contributing variable to the canonical correlation is identified as those that (1) have a high absolute value of the canonical weight assigned to that variable; and (2) have a high absolute ‘canonical loading’ value, which is its correlation with the corresponding canonical variate (e.g. measuring the correlation between $x_{i}$ and *CX* or $y_{j}$ and *CY*). Intuitively, the first measures how much a variable contributes to the canonical variate, the second measures how the change in a variable is reflected in the change of the canonical variate.

### Network analysis

We applied network analysis to individual disease communities to investigate the relationships and flows of information within the communities. Given a set of tweets, we identify the authors of the tweets and plot authors as nodes on a graph. Then for each tweet that mentions other users, an edge is established between the author of the tweet and each mentioned user in the tweet, or the source user in case of a retweet, and target user in case of a reply. These formed the adjacency matrix in the graph. A self-loop edge was created for all tweets that do not interact with other users. We used NodeXL for this part of analysis. The graphs were directed, and the graph’s vertices were all grouped by cluster utilising the Clauset–Newman–Moore cluster algorithm, and the graph was laid out using the Harel–Koren fast multiscale layout algorithm.

## Results

**Fig. 1:**
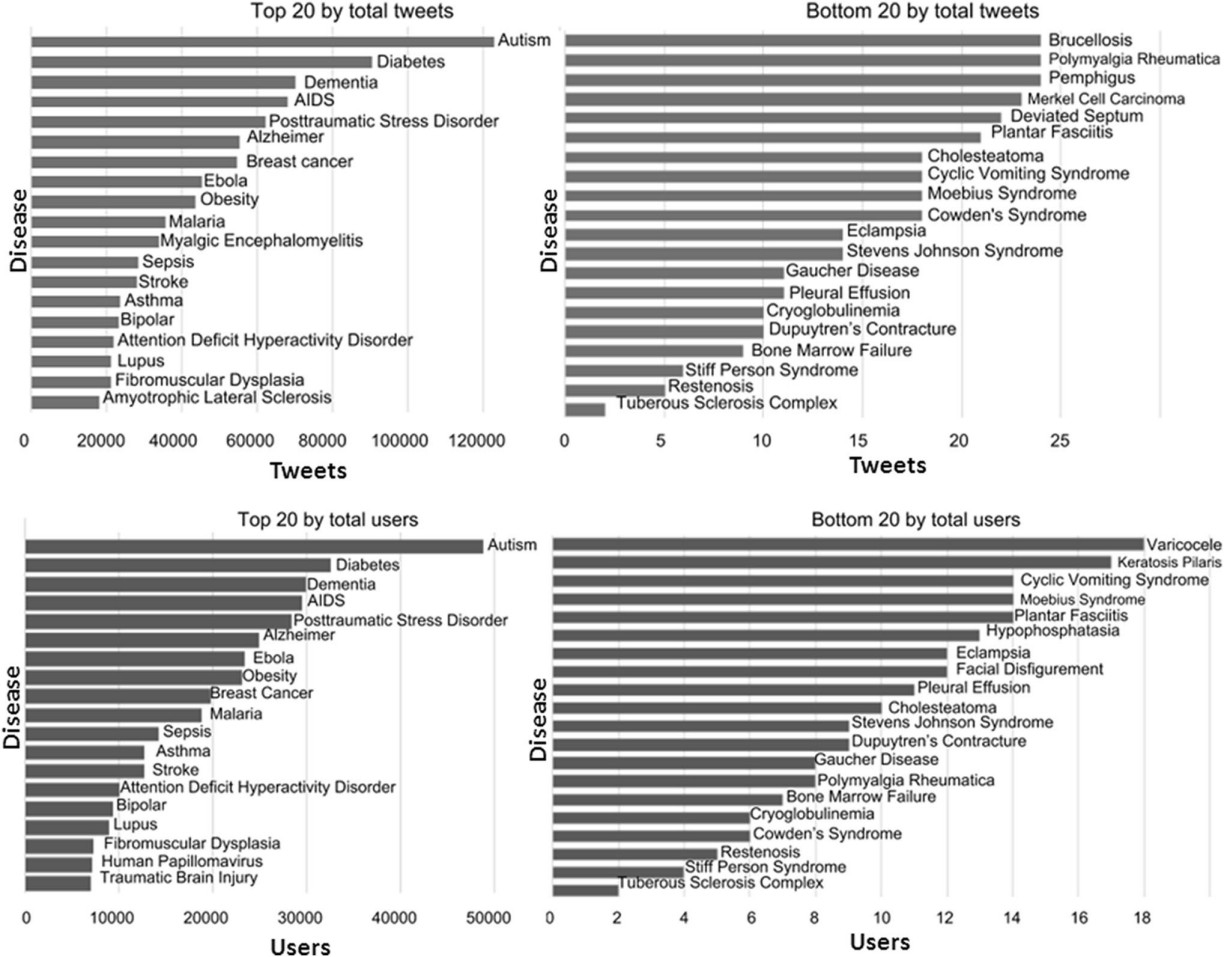
Top 20 and bottom 20 communities ranked by total tweets or users from our sample of worldwide tweets in 2018

**Fig. 2:**
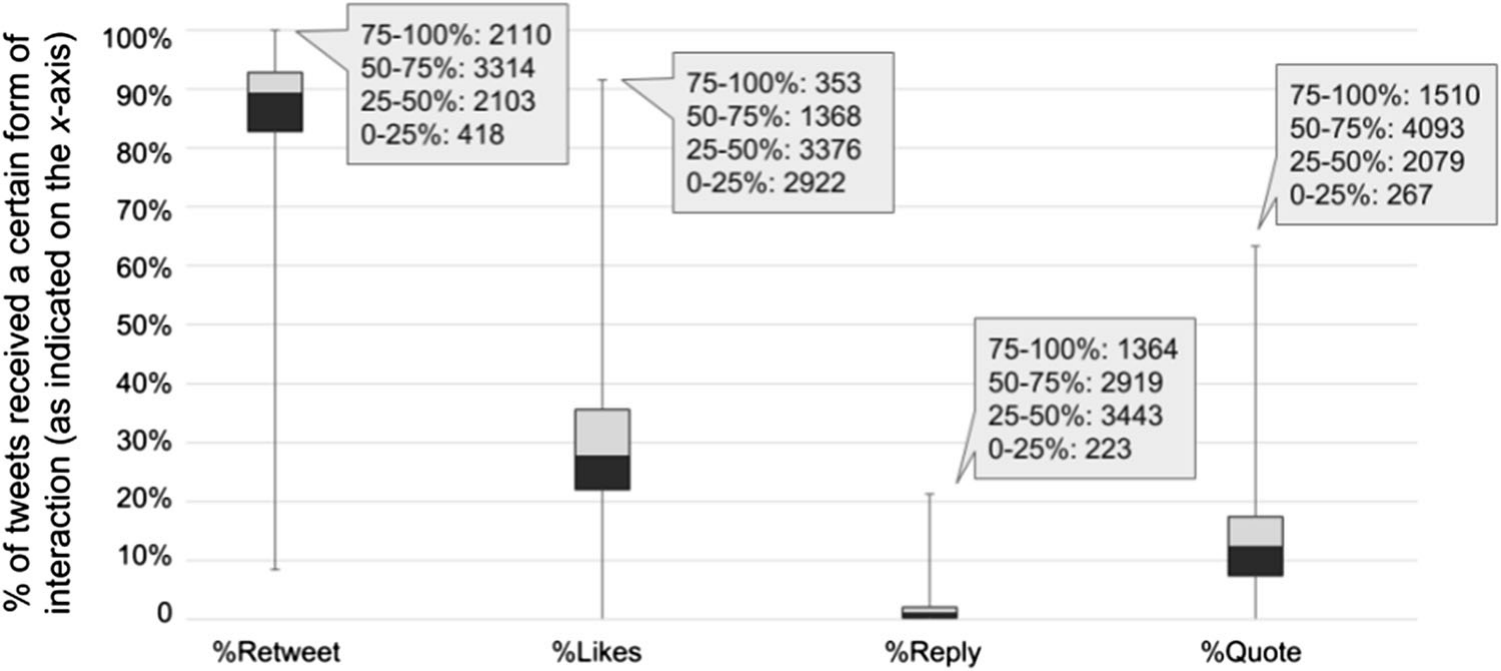
Distribution of communities over different quartiles of %Retweet, %Likes, %Reply and %Quote based on our sample of worldwide tweets in 2018 (numbers inside the call out boxes indicate the average number of users for communities within that quartile)

Due to space constraints, in Fig. 1 we show only the top and bottom 20 communities ranked by their sizes in terms of either total tweets or users. Figure 2 shows the distribution of communities by different variables of engagement. The boxes and whiskers show the range of values of a variable within each quartile, while the callout boxes show the average number of users for each quartile. For example, in terms of %Likes, on average there are 353 users in the 75–100% quantile, which has a %Likes value of 35–92%.

**Fig. 3:**
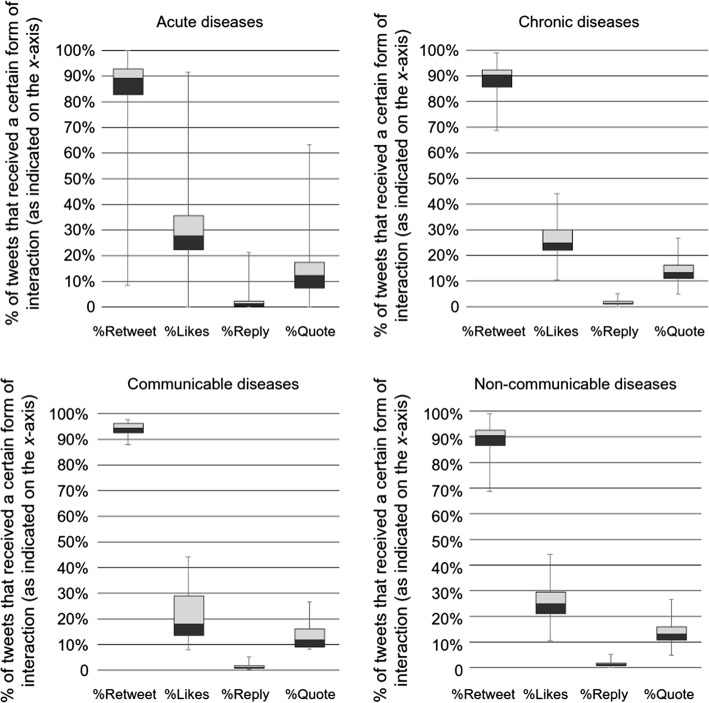
Distribution of communities within subgroups of diseases over different quartiles of %Retweet, %Likes, %Reply and %Quote based on our sample of worldwide tweets in 2018 (numbers inside the call out boxes indicate the average number of users for communities within that quartile)

Further, we selected top 100 diseases either by users or tweets and identified subgroups that are communicable (16), non-communicable (84), acute (20) and chronic (79) diseases. Diseases that can be either communicable or non-communicable (e.g. colitis) and either acute or chronic (e.g. kidney disease) were excluded. Then we plotted boxes and whiskers charts for each subgroup to compare their engagement in Fig. 3.

**Fig. 4:**
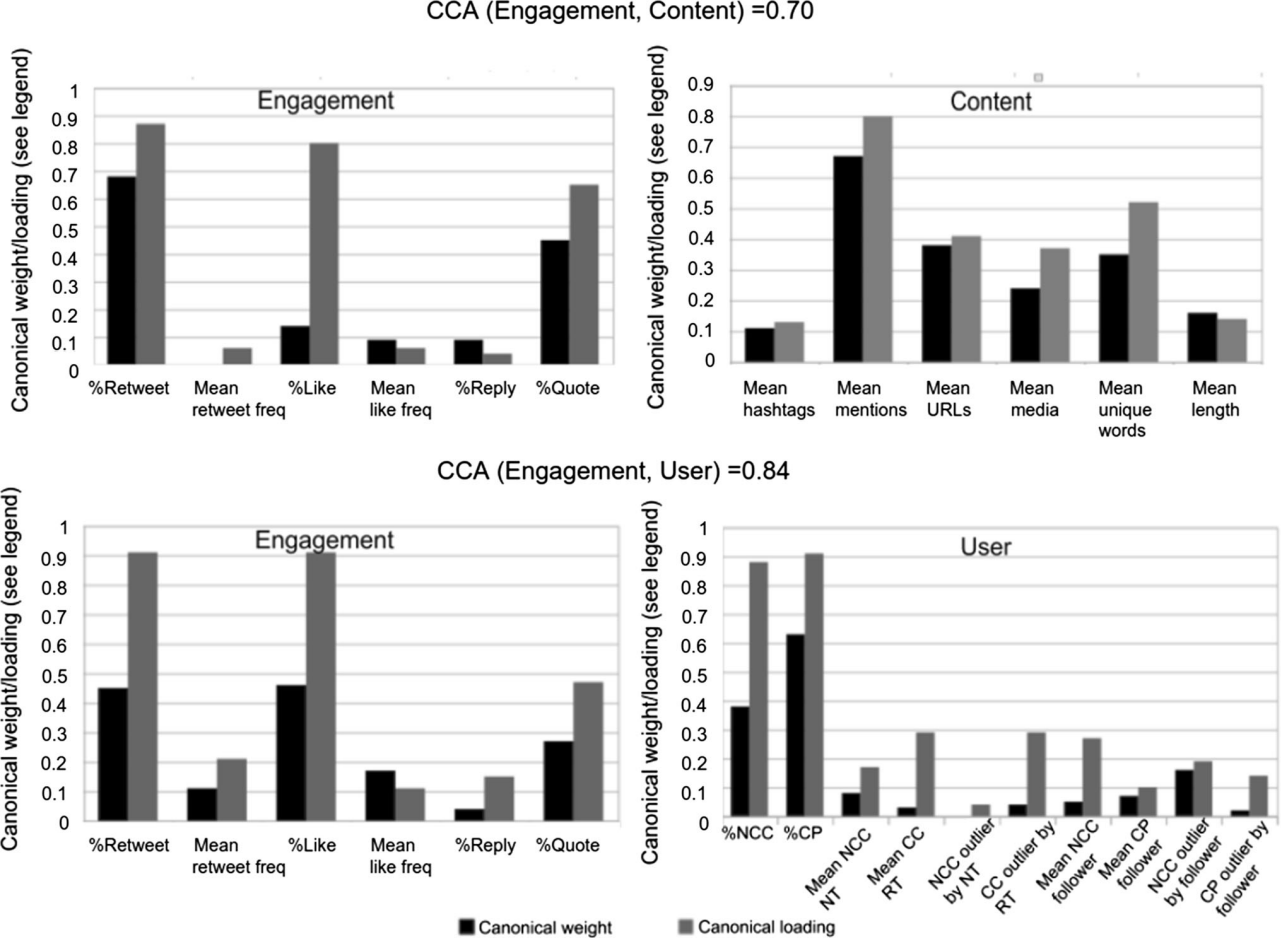
Canonical correlation analysis (CCA) applied to content or user variables against engagement variables, based on our sample of worldwide tweets in 2018 (variables with higher values from each facet potentially have a stronger dependence on each other)

Our pairwise correlation analysis did not identify potentially interesting dependences between engagement variables and the other two facets. The pairs with a score of at least 0.5 were: 0.5 for (%Like, mean unique words), 0.67 for (%Retweet, %NCC), 0.73 for (%Retweet, %CP), 0.74 for (%Retweet, %NCC) and 0.66 for (%Retweet, %CP). Figure 4 shows the canonical correlation between user or content facet with engagement facet, as well as the canonical weights and loadings for each variable of a facet. Variables with a high weight or loading value (or both) are considered ‘important contributor’ to the correlation between the two facets.

Our network analysis selected 12 diseases or disease groups based on the criteria before:

- *Obesity, AIDS and cancer* (combining all kinds of cancers). These are listed by Centers for Disease Control and Prevention as top 10 important health issues affecting the USA
- *Autism, diabetes, dementia, posttraumatic stress disorder* that are among the top five communities by total tweets or users
- *Gout*, with the highest %Like (60%); *sickle cell disease*, with the highest %Retweet (98%); *narcolepsy*, with the highest %Reply (15%) and %Quote (31%); and *chikungunya disease*, with the highest mean followers (by either NCC or CP)
- The combined groups of *Crohn’s disease, colitis and inflammatory bowel disease* (*CC*-*IBD*), as they have a high overlap in terms of users (29–44%)

We show the network structure of these sample communities in Fig. 5 in electronic supplementary material.

## Discussion

### Comparing communities with the multifacet, multivariate model

Among the top communities by tweets shown in Fig. 1, myalgic encephalomyelitis (ME) was particularly interesting, as it was only ranked at 46th by users (approx. 4300), but generated a significant amount of content (tweets). This was potentially due to ME Awareness Day populating the sample when data was collected. It is also important to note that the sizes of the communities did not always correspond to our other definitions of the ‘seriousness’ of the disease. For example, chronic obstructive pulmonary disease (COPD) and tuberculosis were the third and tenth causes of death in the world (WHO 2018), but they each had less than 3000 users and were 46th and 73rd by this standard. Prostate cancer (outside of the top 20) has already overtaken breast cancer to become the third cancer cause of death in countries such as the UK (The Guardian 2018), but it was only about 1/4 the size of the breast cancer community by users or 1/6 by tweets. Many of the smallest communities were related to rare diseases, such as stiff-person syndrome. From a public health perspective, this highlights the needs and potential of improving awareness for certain diseases on social media.

Figure 2 shows retweet to be the most common way of interaction compared to likes, reply and quote; however, it also seems to be the most indifferent measure as more than 75% of the communities had over 80% of their tweets retweeted. Reply, on the other hand, was very rare, perhaps because tweets that actually needed a reply were minority. An interesting pattern is that except for retweets, the communities that had a high engagement level by the other three measures appeared to be smaller in size. In terms of likes, the average number of users in the communities located in the upper quartile of the distribution (75–100%) was only 353, which is significantly smaller compared to other parts of the distribution. These communities have between 35 and 92% of their tweets liked (as indicated by the *y*-axis). In fact, none the top 20 communities (by users) were in the upper quartile in terms of %Like. Although we also compared engagement patterns within different subgroups of diseases in Fig. 3, we could not identify notable difference from the overall pattern.

### Correlation analysis

Among the five notable pairwise correlations described before, the first (%Like, mean unique words) seems to suggest that users preferred tweets that were longer, but also used a diverse set of words, possibly because they were more informative. However, the correlation score was not strong enough to assert such a link. The others were arguably less interesting: if there were a larger percentage of users that engaged in creating or propagating content, we could normally expect a larger percentage of tweets to be retweeted or liked. In fact, the dependence between engagement variables and other facets may be so complex that pairwise correlation is inadequate to explain.

In terms of CCA, as Fig. 4 shows, a correlation of 0.70 between content and engagement variables. The highest contributing variable of content was mean mentions, which had both a high canonical weight (0.67) and loading (0.80, both on a scale between 0 and 1). Other arguably important but less significant variables were mean URLs and unique words, which had weights of 0.34 and 0.38, but they correlated less (i.e. loading) to the content canonical variate. For engagement, %Retweet had both the highest weight (0.67) and loading (0.87). %Like and %Quote both correlated highly with the engagement canonical variate, but had lower weight. Overall, this potentially suggests that targeted tweets (using mentions) containing rich use of URLs, diverse words and possibly media, were most likely to be retweeted, quoted or liked. These types of tweets addressed to specific users are likely to provide rich contextual information. Interestingly, the use of hashtags and long tweets that possibly contained repetitive words was not helpful.

The correlation between engagement and user variables was even higher, at 0.84. Having a high percentage of active users, either creating new (%NCC) or propagating existing content (%CP) potentially increased retweets, likes and quotes. Notice also the notable weights of %NCC-outliers by followers, which could suggest that having influential users (those with a significantly high number of followers) to act as ‘advocate’ to actively tweet in a community can also be important for engagement. This finding could be of potential interest to public health authorities who could utilise marketing tactics such as identifying influential users for information propagation.

### Network analysis

To interpret the networks shown in Fig. 5 in electronic supplementary material, we follow the guideline from Smith et al. (2014). The network structures appeared to look similar for all conditions except diabetes. Relating to some of our selection criteria discussed before, it appears that the network structure has no correlation with some of the variables used for selection. For each health condition, we can identify several main groups of users forming dense clusters representing small to mid-size communities, potentially discussing subtopics related to the conditions. We can also see an isolates group (rectangle or square shaped group) which highlights users that were rarely or sometimes not connected to each other. Many of these were users that sent single tweets offering their opinion and/or tweeted news articles but received little or no interaction.

Among those densely connected clusters for each condition, we can identify two patterns. The first includes several large ‘broadcast’ clusters featuring distinctive hub and spoke structure where the audience was often connected to one or a few ‘central’ nodes without connecting to one another. This suggests that certain Twitter users would drive the conversation and certain tweets would attract a larger proportion of likes and retweets. For example, most of the large clusters from narcolepsy, chikungunya and gout communities feature this pattern. The second includes a large number of smaller groups that look like ‘bazaars’ featuring multiple centres of activity. This is called ‘community clusters’ and indicates smaller pockets of discussion and mutual information exchange related to subtopics. Examples include the smaller clusters in the Autism and Cancer communities.

Interestingly, the Twitter community of the diabetes network seemed slightly different. On the one hand, there are more community clusters; on the other hand, these are much smaller and non-dominating. This potentially indicates that diabetes has more community-based discussions. Diabetes organisations could seek to share engaging and/or informative content in order to develop a broadcast structure. These are often observed in Twitter accounts belonging to influential users who had a special interest in the health condition and actively provide support and raise awareness. They are beneficial because they support the cascading of information in the network.

It must be noted that our network analysis is based on a sample of the entire dataset, which means that there may be a slight variance in our outputs to the full network of health conditions analysed. Nevertheless, we have found that the network structures are largely consistent in many conditions.

### Implication of this research

One of the key challenges for public health authorities and policy makers is where and how to intervene. This study would enable an evidence-based approach to identify online health-related communities that may potentially benefit from intervention, based on the observed patterns of engagement. It also identifies factors that potentially affect engagement, thus allowing effective intervention measures to be developed. For researchers and practitioners, our findings would enable them to target conditions with greater potential for value.

### Limitations of the work

A limitation of our study is the lack of qualitative analysis and future work will seek to analyse topics of content shared within different communities and/or the types of stakeholders involved, in order to understand if such factors may affect engagement. Furthermore, identifying influential users in the social network analysis may reveal their roles in the promotion and growth of a community. A further limitation is the incompleteness in the list of health conditions represented in our study. While we opted to focus our study on the standard approach of following hashtags compiled by Symplur for data collection, it is known that the list is constantly growing and revised. As a result, a number of health conditions may not be covered. An alternative could be to use instead, a list of disease keywords for data collection. Future work will address both issues.

### Conclusion

This work compared the information sharing within Twitter user communities in over 300 different health conditions by analysing 1.5 million tweets generated by over 450,000 users during a 1-month time period. We proposed a multivariate model to quantify the engagement, user and content characteristics of each community and conducted correlation and network analysis to discover patterns of user activities. Our results are likely to be of interest to public health authorities interested in the potential of Twitter to raise awareness of public health.

## Electronic supplementary material

Below is the link to the electronic supplementary material.

Supplementary material 1 (DOCX 1642 kb)
