# Supplementary material for: A comparison of information sharing behaviours across 379 health conditions on Twitter
Source: Int J Public Health. 2018 Dec 26;64(3):431–40. doi: 10.1007/s00038-018-1192-5 (PMC6451705; doi:10.1007/s00038-018-1192-5)

**International Journal of Public Health**

A Comparison of Information Sharing Behaviours across 379 Health Conditions on Twitter

**Fig. 5** Social Network Graphs of 12 different health conditions on Twitter, based on our sample of worldwide tweets in 2018 (each dot represents a Twitter user and each line represents an edge)


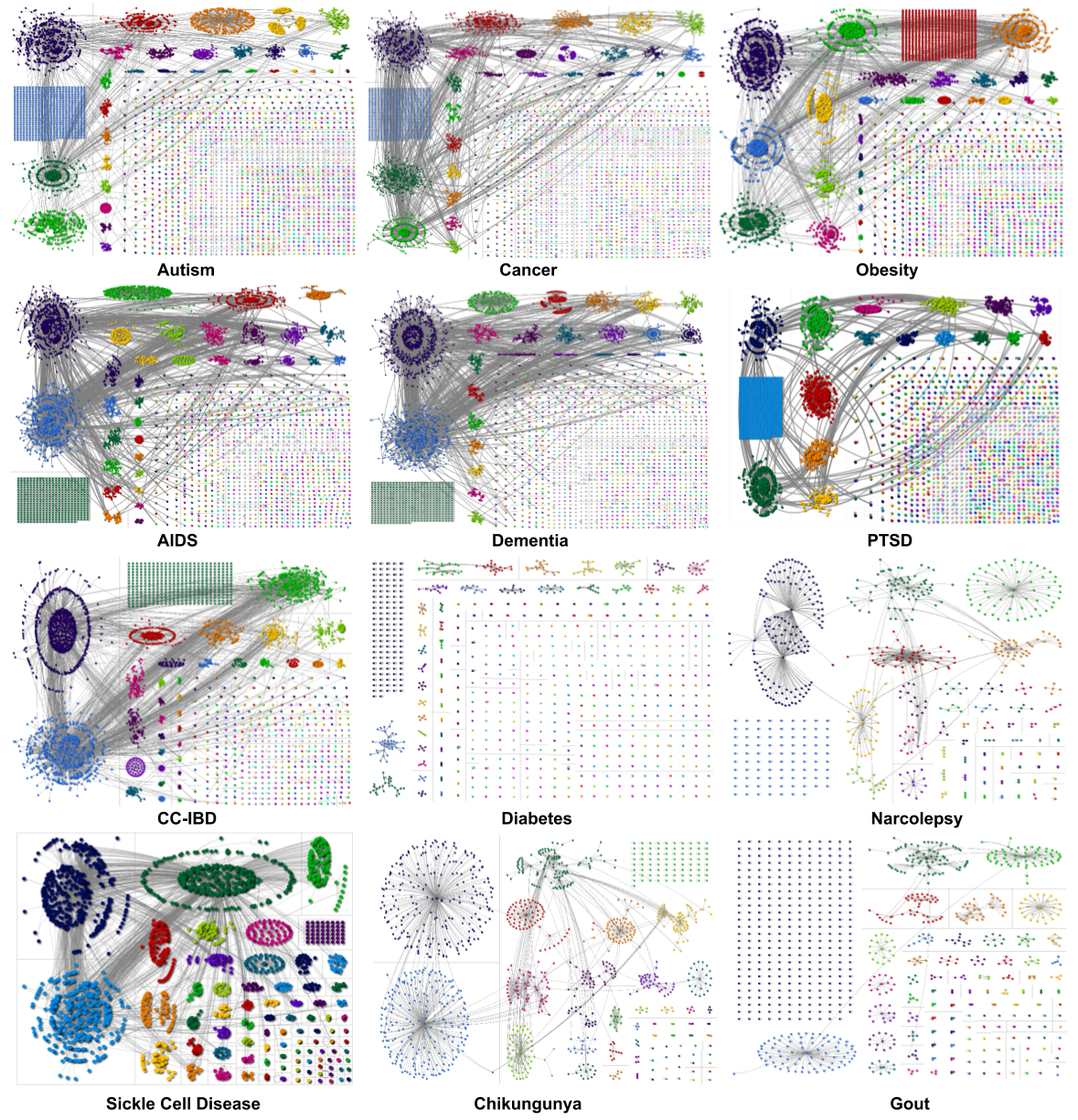

Supplement: Supplementary file 1 — Supplementary material 1 (DOCX 1642 kb) [file 38_2018_1192_MOESM1_ESM.docx]
